# Effects of high incubation temperature on tight junction proteins in the yolk sac and small intestine of embryonic broilers

**DOI:** 10.1016/j.psj.2023.102875

**Published:** 2023-06-17

**Authors:** M. Jia, K.L. Reynolds, E.A. Wong

**Affiliations:** School of Animal Sciences, Virginia Tech, Blacksburg, VA 24061, USA

**Keywords:** tight junction protein, yolk sac, small intestine, occludin, junctional adhesion molecule

## Abstract

During the transition from incubation to hatch, the chicks shift from obtaining nutrients from the yolk sac to the intestine. The yolk sac tissue (**YST**) and small intestine serve as biological barriers between the yolk or gut contents and the blood circulation. These barriers must maintain structural integrity for optimal nutrient uptake as well as protection from pathogens. The objective of this study was to investigate the effect of high incubation temperature on mRNA abundance of the tight junction (**TJ**) proteins zona occludens 1 (**ZO1**), occludin (**OCLN**), claudin 1 (**CLDN1**), and junctional adhesion molecules A and 2 (**JAMA, JAM2**) and the heat shock proteins (**HSP70** and **HSP90**) in the YST and small intestine of embryonic broilers. Broiler eggs were incubated at 37.5°C. On embryonic day 12 (**E12**), half of the eggs were switched to 39.5°C. YST samples were collected from E7 to day of hatch (**DOH**), while small intestinal samples were collected from E17 to DOH. The temporal expression of TJ protein mRNA from E7 to DOH at 37.5°C and the effect of incubation temperature from E13 to DOH were analyzed by one-way and two-way ANOVA, respectively and Tukey's test. Significance was set at *P* < 0.05. The temporal expression pattern of ZO1, OCLN, and CLDN1 mRNA showed a pattern of decreased expression from E7 to E13 followed by an increase to DOH. High incubation temperature caused an upregulation of ZO1 and JAM2 mRNA in the YST and small intestine. Using in situ hybridization, OCLN and JAMA mRNA were detected in the epithelial cells of the YST. In addition, JAMA mRNA was detected in epithelial cells of the small intestine, whereas JAM2 mRNA was detected in the vascular system of the villi and lamina propria. In conclusion, the YST expressed mRNA for TJ proteins and high incubation temperature increased ZO1 and JAM2 mRNA. This suggests that the TJ in the vasculature of the YST and intestine is affected by high incubation temperature.

## INTRODUCTION

The embryonic chick is dependent upon a functional yolk sac and the development of the small intestine for the uptake of nutrients (Lillburn and Loefler, 2015; Wong and Uni, 2021). Environmental factors such as suboptimal incubation temperature has been shown to play an important role in embryonic chick growth, with an elevated incubation temperature (>37.8°C) negatively affecting development of the embryo (van der Wagt et al., 2020). However, only a few studies have shown the effect of incubation temperature on the development of these 2 important organs.

The yolk sac tissue (**YST**) is an extra-embryonic membrane that is a multifunctional organ involved in nutrient uptake, immunity, hematopoiesis, and other functions (Yadgary et al., 2011; Guedes et al., 2014; Zhang and Wong, 2017, 2019; Wong and Uni, 2021). The YST plays an important role in protecting the embryo from pathogens by acting as a physiological barrier and by expressing avian β-defensin 10 (Zhang and Wong, 2019). Because the YST is derived from the small intestine, structural and functional properties of the YST are similar to the small intestine (Starck, 2021; Wong and Uni, 2021). Both the YST and the intestinal villi are lined with a single layer of functional epithelial cells. Mobbs and McMillan (1979) showed with electron microscopy the presence of tight junctions (**TJ**) in the *area pellucida* and *area vasculosa* of the YST. The TJ seals the endodermal epithelial cells and maintains the integrity of the endoderm to prevent the intercellular passage of the yolk. Donaldson et al. (1990) further demonstrated that TJ formed between yolk sac epithelial cells in cell culture. Expression of TJ mRNA in the YST, however, has not been reported.

The TJ is one of the elements of the epithelial junctional complex that is located in the apical lateral membrane of intestinal cells (Farquhar and Palade, 1963; Zihni et al., 2016). The TJ forms a physiological barrier that maintains the integrity of the epithelial cell layer and plays an essential role in the regulation of paracellular transport of ions and water (Van Itallie and Anderson, 2004). A series of transmembrane proteins, including occludin (**OCLN**), claudins (**CLDN**), and junctional adhesion molecules (**JAM**) (Zihni et al., 2016; Awad et al., 2017) are part of the TJ. There are also cytosolic proteins, such as zonula occludens 1 (**ZO1**), that link membrane components with the actin cytoskeleton.

Heat stress during incubation dramatically decreased hatchability and performance in the posthatch stages of broiler chickens (Narinç et al., 2016). The effect of heat stress on TJ mRNA expression has been examined in the small intestine of posthatch chickens (Song et al., 2014; Zhang et al., 2017), but not in the YST or embryonic intestine. Thus, the objective of this study was to examine the effect of high incubation temperature on mRNA abundance of the tight junction proteins ZO1, OCLN, CLDN1, JAMA, and JAM2 as well as the heat shock proteins (**HSP**) 70 and 90 in the YST and small intestine of embryonic broiler chickens.

## MATERIALS AND METHODS

### Egg Incubation and Tissue Collection

***Experiment 1***. Cobb 500 broiler eggs were obtained from a local commercial hatchery and incubated at 37.5°C. All animal procedures were approved by the Virginia Tech Institutional Animal Care and Use Committee. At embryonic (**E**) day 7, E9, and E11, 6 eggs were randomly selected and the embryo was euthanized by cervical dislocation for collection of YST. At E12, eggs were candled and infertile eggs were removed. Fertility was 84%. One set of fertile eggs was transferred to an incubator set at 39.5°C and another set was kept at 37.5°C. At E13, E15, E17, E19, and day of hatch (**DOH**), 6 eggs from each treatment were randomly selected and the embryo was euthanized by cervical dislocation. A piece of YST (∼1 cm^2^) from the *area vasculosa* was collected at these time points. At E17, the entire small intestine was collected, due to the softness of the tissue, whereas at E19 and DOH, the jejunum was collected. The YST and small intestine were rinsed in cold 1 × PBS to remove the residual yolk and intestinal contents, respectively. Pieces of YST and small intestine were minced, rapidly frozen on dry ice and stored at −80°C for subsequent gene expression analysis. Yolk-free body weights (**YFBW**) of sampled embryos were collected from E7 to E19 and chick BW was collected at DOH.

***Experiment 2.*** To further investigate the temporal pattern of gene expression in the YST using in situ hybridization (**ISH**), a second experiment was conducted at only 37.5°C. Cobb 500 eggs from a local hatchery were incubated at 37.5°C. Fertility was 97% and hatchability was 100%. At E7, E9, E11, E13, E15, E17, E19, and DOH, 6 eggs were randomly selected and a piece of YST (∼1 cm^2^) was collected from the *area vasculosa* of euthanized chicks. For gene expression, YST samples were rinsed free of residual yolk, frozen and stored at −80°C. For ISH, pieces of YST from each chick were fixed in 10% neutral-buffered formalin for 24 h at room temperature, transferred to 70% ethanol for 24 h at room temperature, and then stored in fresh 70% ethanol at 4°C. These samples were shipped to StageBio (Mount Jackson, VA) for paraffin embedding.

### RNA Extraction and Gene Expression Analysis

Frozen YST (*n* = 6) from E7 to DOH and intestinal samples (*n* = 6) from E17 to DOH were homogenized in TRI Reagent (Molecular Research Center, Inc., Cincinnati, OH) with a Tissue Lyser II (Germantown, MD). Total RNA was extracted following the instructions of the Direct-zol RNA MiniPrep kit (Zymo Research, Irvine, CA). The concentration and purity of RNA were assessed using a Nanodrop 1000 spectrophotometer (Thermo Fisher Scientific, Pittsburgh, PA). cDNA was synthesized starting with 1 μg total RNA using the High-Capacity cDNA Reverse Transcription Kit (Thermo Fisher Scientific), and then diluted 1:20 with DEPC water for real time quantitative PCR (**qPCR**). cDNA was diluted 1:200 for qPCR analysis of 18S ribosomal RNA (**rRNA**) as a reference gene. Each qPCR reaction contained 5 μL of Fast SYBR Green Master mix (Thermo Fisher Scientific), 1.5 μL of diluted cDNA, 1 μL of forward primer (5 μM), 1 μL of reverse primer (5 μM), and 1.5 μL of DEPC water. qPCR was performed using an Applied Biosystems 7500 Fast Real-time PCR system (Thermo Fisher Scientific) with the default fast program: 95°C for 20 s, 40 cycles of 90°C for 3 s and 60°C for 30 s. Each sample was run in duplicate. The mRNA abundance of selected TJ proteins including ZO1, OCLN, CLDN1, JAMA, JAM2 were determined using the primers shown in Table 1. JAM2 is equivalent to mammalian JAMB as reported by Goo et al. (2019). To confirm that incubation at 39.5°C was having an effect, expression of HSP70 and HSP90 were also analyzed from E13 to DOH. The stability of 5 reference genes including ribosomal protein subunit P0 (**RPLP0)** and ribosomal protein subunit P1, glyceraldehyde-3-phosphate dehydrogenase, β-actin, and 18S ribosomal RNA (**rRNA**) were analyzed for stability using RefFinder (Xie et al., 2012). Due to their higher stability, only RPLP0 and rRNA were used as reference genes. The geometric mean of the Ct values for RPLP0 and rRNA was used as the Ct value of the reference gene in the YST and small intestine for both Experiment 1 and 2. Relative gene expression levels were calculated using the 2^−ΔΔCt^ method (Schmittgen and Livak, 2008). For the temporal gene expression analysis of the YST from E7 to DOH, the average ΔCt value at 37.5°C for the E7 timepoint was used as the calibrator for incubation at 37.5°C in both Experiment 1 and 2. For the effect of incubation temperature, the average ΔCt value at 37.5°C for the E13 time point was used as the calibrator for the YST and the average ΔCt value at 37.5°C for the E17 time point was used as the calibrator for the small intestine.

**Table 1:** Primers for qPCR.

Gene abbreviation	Gene name	Forward primer (5′–3′)	Reverse primer (5′–3′)	Amplicon size (bp)	Accession number
ZO1^1^	Zonula occludens-1	TGTAGCCACAGCAAGAGGTG	CTGGAATGGCTCCTTGTGGT	98	XM_015278975.2
OCLN^2^	Occludin	TCATCGCCTCCATCGTCTAC	TCTTACTGCGCGTCTTCTGG	240	NM 205128.1
CLDN1^2^	Claudin-1	TGGAGGATGACCAGGTGAAGA	CGAGCCACTCTGTTGCCATA	115	NM 001013611.2
JAMA^3^	Junctional adhesion molecule A	GAAAACCAACCCGTGGACAT	GGAAGAGCCCTTCTGGAACTT	90	NM_001083366.1
JAM2^4^	Junctional adhesion molecule 2	AGCCTCAAATGGGATTGGATT	CATCAACTTGCATTCGCTTCA	59	NM_001397141.1
HSP70^5^	Heat shock protein 70	TCGGCCGCAAGTATGATGA	CGGAAGGGCCAGTGCTT	58	NM_001006685.1
HSP90^5^	Heat shock protein 90	GCAGCAGCTGAAGGAATTTGA	GGAAGCTCTAAGCCCTCTTTTGT	66	NM_001109785.1
RPLP0^6^	Ribosomal protein lateral stalk subunit P0	GCGATTGCTCCCTGTGATG	TCTCAGGTCCGAGACCAGTGT	59	NM_204987.2
rRNA	Ribosomal RNA	CCGTCGTAGTTCCGACCATAA	GCGGGTCATGGGAATAACG	65	XR_006936397.1

Primers were from

1 Li et al. (2015).

2 Shao et al. (2013).

3 Zhang et al. (2017), JAMA is also known as F11 receptor.

4 Chen et al. (2015), JAM2 is the same as JAMB.

5 Reynolds (2019).

6 Zhang and Wong (2019).

### RNAscope In Situ Hybridization

Three of the 6 formalin-fixed paraffin-embedded tissue blocks were randomly chosen and cut into 5 μm sections using a microtome (Microm HM 355S; Thermo Fisher Scientific). The sections were mounted on Superfrost-Plus glass slides (Electron Microscopy Sciences, Hatfield, PA). The RNAscope ISH procedure was performed using the RNAscope 2.5 HD Assay-RED and custom probes for chicken OCLN (Accession number: NM_205128.1), JAMA (Accession number: NM_001083366.1), and JAM2 (Accession number: NM_001397141.1) according to the instructions of Advanced Cell Diagnostics (Newark, CA). Slides were counterstained with 50% hematoxylin Gill I (Sigma-Aldrich, St. Louis, MO). Images were captured using fluorescence with a Nikon Eclipse 80i microscope and Nikon DS-Ri1 or DS-Ri2 cameras.

### Statistical Analysis

Statistical analysis was performed using JMP Pro 15 (SAS Institute Inc., Cary, NC). Differences in YFBW within day between the 37.5°C and 39.5°C incubation temperatures from E13 to DOH were analyzed by *t* test. Differences of gene expression in the YST between ages from E7 to DOH at the 37.5°C incubation temperatures were analyzed by one-way ANOVA and Tukey's test. Differences of gene expression in the YST (E13 to DOH) and small intestine (E17 to DOH) between ages or incubation temperatures were analyzed by two-way ANOVA and Tukey's test. qPCR data were log10 transformed prior to statistical analysis for the YST, because the data did not fit a normal distribution. Significance was set at *P* < 0.05.

## RESULTS

**Figure 1:**
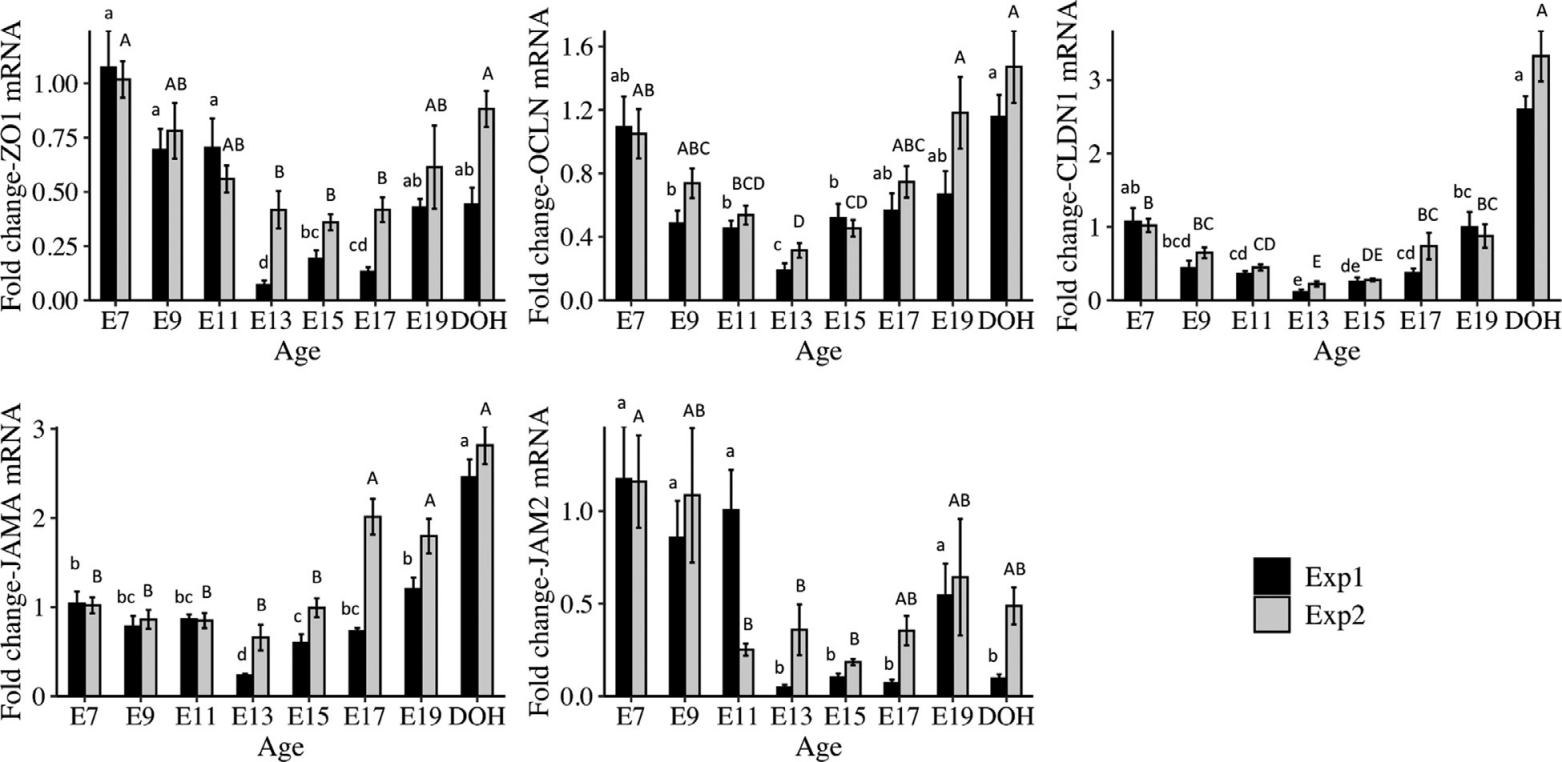
mRNA expression profiles of tight junction proteins in the yolk sac tissue of embryos and day of hatch chicks. Two replicate experiments Experiment 1 (Exp1) and Experiment 2 (Exp2) were conducted. Eggs were incubated at 37.5°C. Yolk sac tissue samples were collected from 6 chicks from embryonic d 7 (E7) to day of hatch (DOH). Gene expression was determined using relative qPCR (*n* = 6). Bars show mean ± individual SE. Significance (*P* < 0.05) was analyzed by one-way ANOVA and Tukey's test, following log 10 transformation. Different lowercase letters (a–e) indicate a difference between days within Exp1. Different uppercase letters (A–E) indicate a difference between days within Exp2.

The temporal expression patterns of TJ and heat shock protein mRNA in the YST from E7 to DOH at 37.5°C from duplicate experiments are shown in Figure 1. For Experiments 1 and 2, ZO1 mRNA decreased from E7 to E13 through E17 and then increased to DOH. OCLN mRNA decreased from E7 to E13 and then increased to DOH. CLDN1 mRNA decreased from E7 to E13 and E15 and then increased to DOH. JAMA mRNA decreased from E7 to E13 and E15 and then increased to DOH in Experiment 1, but showed low expression from E7 to E15 and then increased to DOH in Experiment 2. JAM2 mRNA decreased from E7 to E13 and remained low to DOH, with a transient increase at E19 in Experiment 1. In Experiment 2, JAM2 mRNA decreased from E7 to E11 and remained unchanged to DOH.

**Figure 2:**
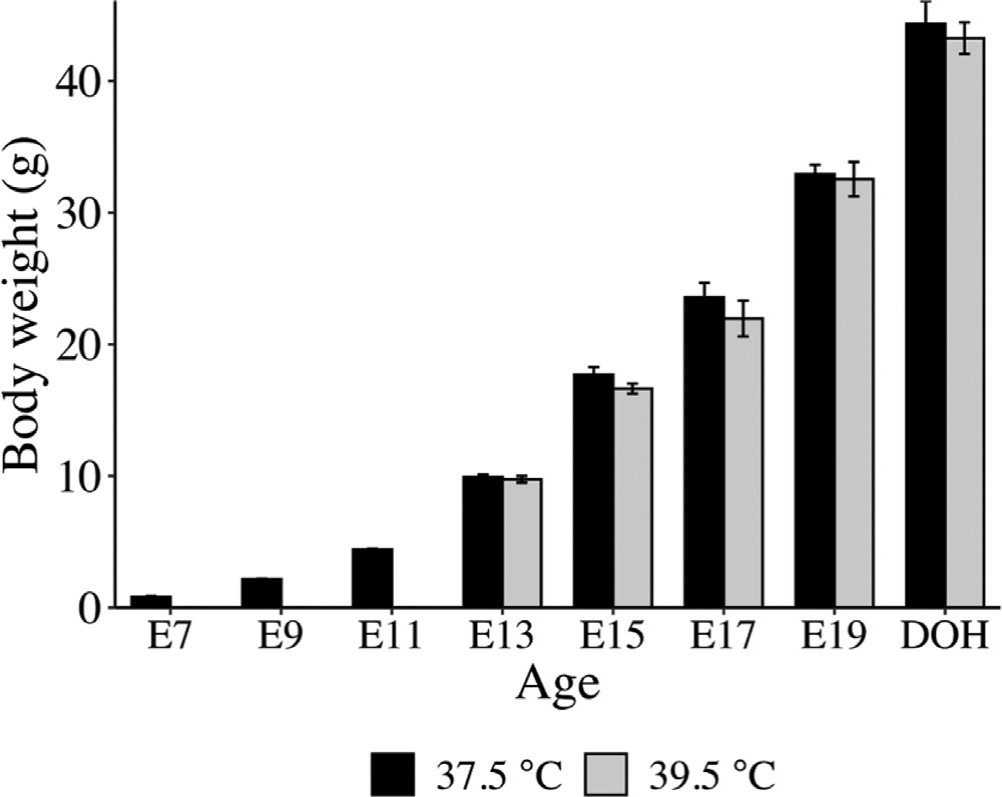
Effect of incubation temperature on body weight of embryos and day of hatch chicks. Eggs were incubated at 37.5°C from embryonic d 0 to 12 (E12). At E12, half of the fertile eggs were transferred to 39.5°C. Yolk-free body weights of embryos from embryonic d 7 (E7) to E19 (*n* ≥ 6) and whole body weights of chicks at DOH (*n* = 6) were measured.

The effect of high incubation temperature on body weights and expression of TJ mRNA in the YST and small intestine was investigated. Incubation at 39.5°C from E12 to DOH did not affect the embryonic YFBW from E13 to E19 or BW at DOH compared to 37.5°C (Figure 2). Hatchability of chicks, however, was dramatically lower at 39.5°C (42%) compared to 37.5°C (93%). On d 21 of incubation, chicks hatched from eggs incubated at 39.5°C were delayed compared to chicks hatched from eggs incubated at 37.5°C.

**Figure 3:**
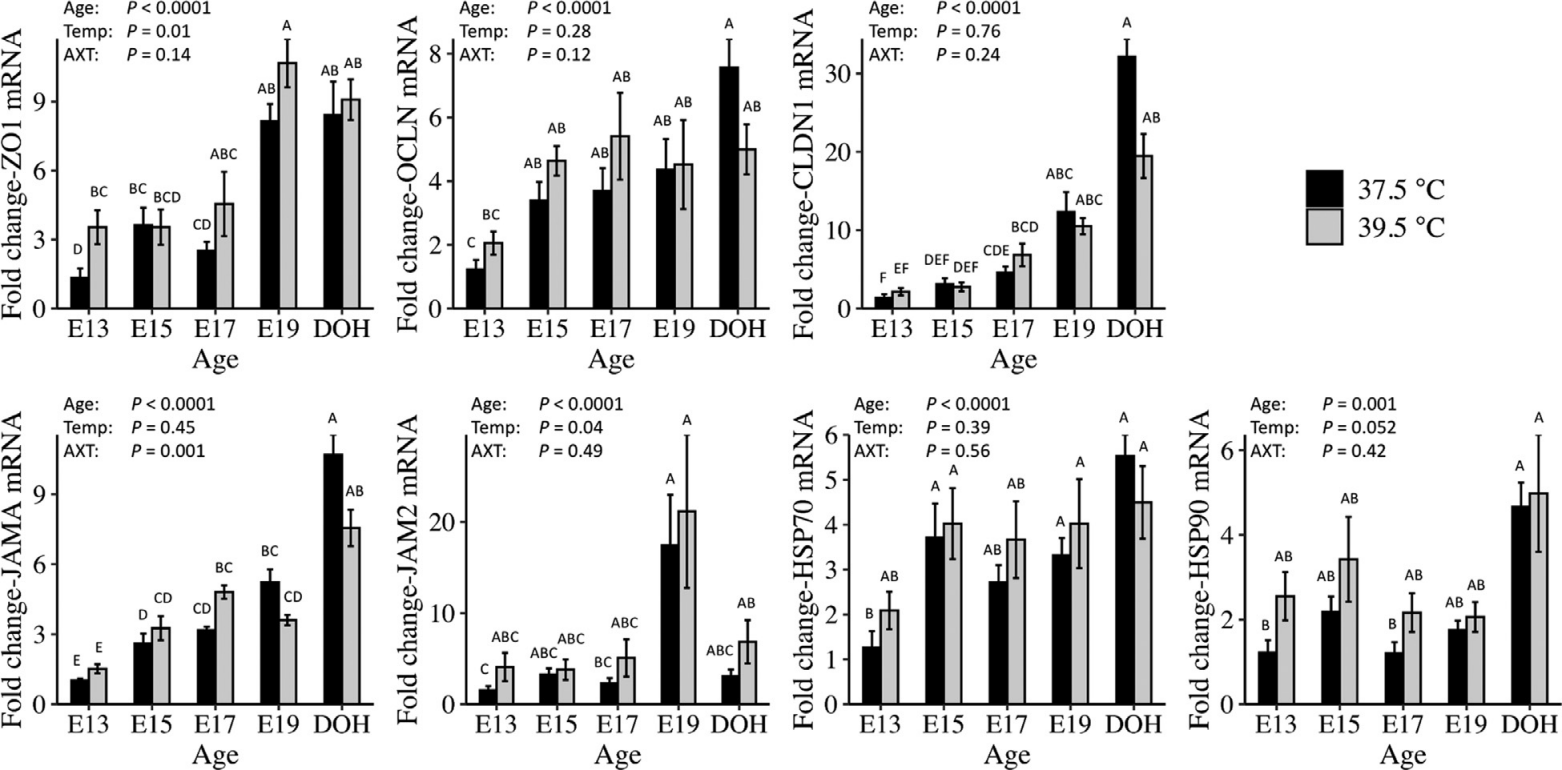
Effect of incubation temperature on mRNA abundance of tight junction and heat shock proteins in the yolk sac tissue of embryos and day of hatch chicks. Eggs were incubated at 37.5°C from embryonic d 0 to 12 (E12). At E12, half of the fertile eggs were transferred to 39.5°C (Experiment 1). Yolk sac tissue samples were collected from 6 chicks per treatment from E13 to day of hatch (DOH). Gene expression was determined using relative qPCR (*n* = 6). Significance (*P* < 0.05) was analyzed by two-way ANOVA and Tukey's test, following log 10 transformation. Bars show mean ± individual SE for each gene. Different letters (A–E) indicate a difference between days or incubation temperature.

Incubation at 39.5°C from E12 to DOH affected mRNA abundance of selected TJ and heat shock proteins compared to 37.5°C in the YST (Figure 3). ZO1 mRNA showed main effects of Age and Temperature. ZO1 mRNA increased from E13 to DOH and was greater at 39.5°C than 37.5°C. OCLN and CLDN1 mRNA both showed main effects of Age, with increases from E13 to DOH. JAMA mRNA showed an Age × Treatment interaction. At 37.5°C, JAMA mRNA increased from E13 to E15, from E15 to E19 and from E19 to DOH. By contrast, at 39.5°C JAMA mRNA increased from E13 to E15, did not change from E15 to E19 and then increased from E19 to DOH. JAM2 mRNA showed a main effect of Age and Temperature. JAM2 mRNA increased from E13 to E19 and was greater at 39.5°C than 37.5°C. For the heat shock proteins, HSP70 and HSP90, there were main effects of Age, with increases from E13 to DOH.

**Figure 4:**
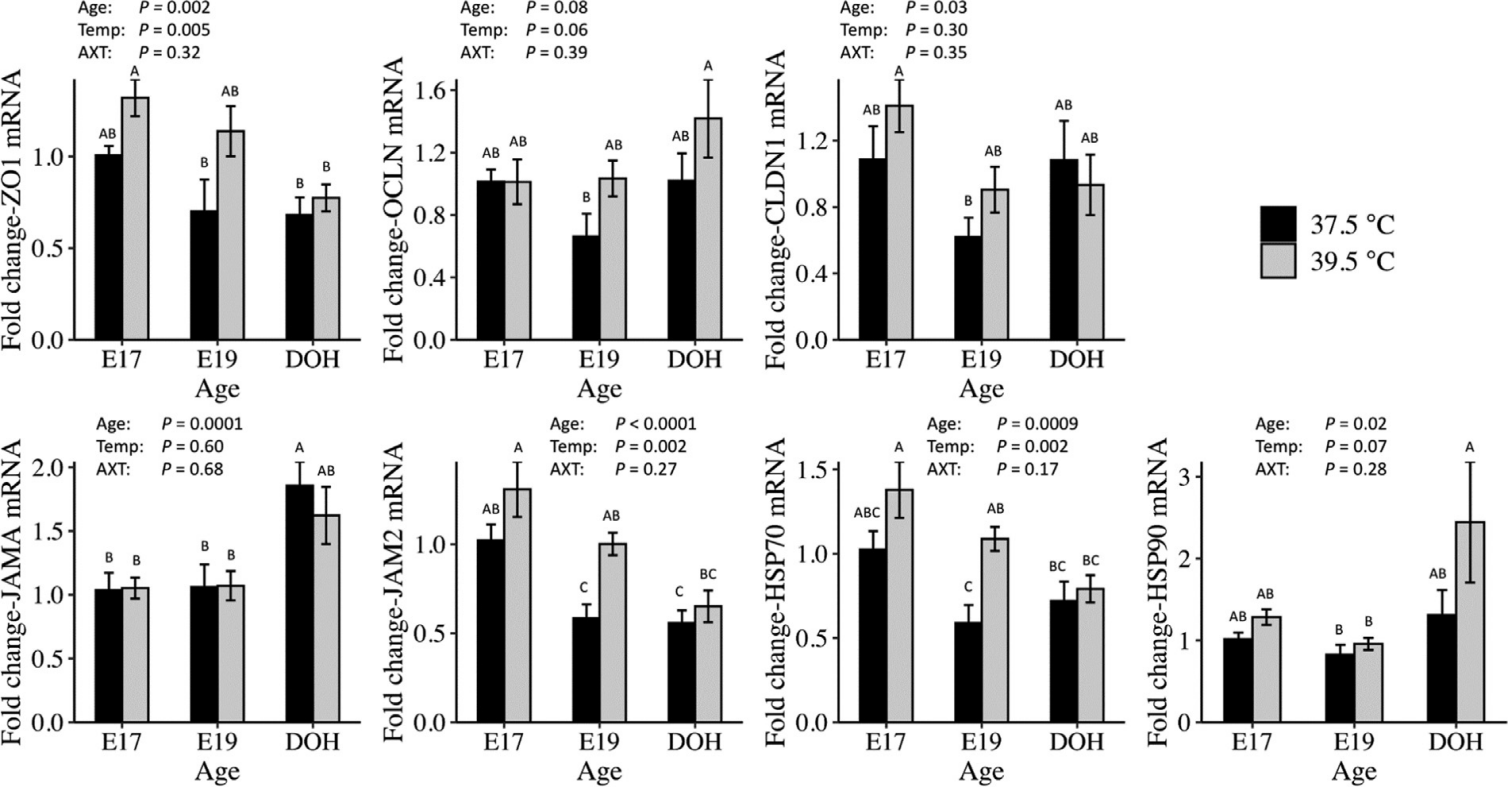
Effect of incubation temperature on mRNA abundance of tight junction and heat shock proteins in the small intestine of embryos and day of hatch chicks. Eggs were incubated at 37.5°C from embryonic d 0 to 12 (E12). At E12, half of the fertile eggs were transferred to 39.5°C (Experiment 1). The whole small intestine was collected from 6 chicks per treatment on E17. The jejunum was collected on E19 and DOH. Gene expression was determined using relative qPCR (*n* = 6). Significance (*P* < 0.05) was analyzed by two-way ANOVA and Tukey's test. Bars show mean ± individual SE. Different letters (A–C) indicate a difference between days or incubation temperature.

High incubation temperature from E12 to DOH also altered mRNA abundance of TJ and HSP proteins in the small intestine (Figure 4). ZO1 mRNA showed main effects of Age and Temperature. ZO1 mRNA decreased from E17 to DOH and was greater at 39.5°C than 37.5°C. OCLN mRNA was not affected by high incubation temperature. CLDN1 and JAMA mRNA both showed main effects of age. CLDN1 mRNA decreased from E17 to E19, while JAMA mRNA increased from E17 to DOH. JAM2 mRNA showed main effects of Age and Temperature. JAM2 mRNA decreased from E17 to DOH and was greater at 39.5°C than 37.5°C. For the heat shock proteins, HSP70 showed both main effects of Age and Temperature. HSP70 mRNA decreased from E17 to DOH and was greater at 39.5°C than 37.5°C. HSP90 mRNA showed a main effect of Age, with an increase from E19 to DOH.

**Figure 5:**
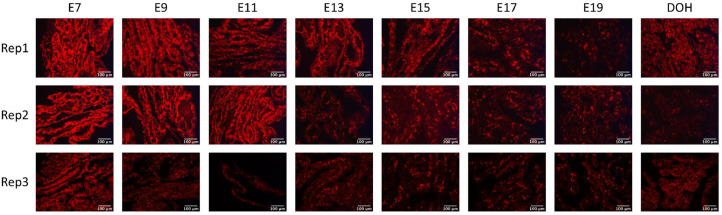
In situ hybridization for occludin mRNA in the yolk sac tissue of embryos and day of hatch chicks. Eggs were incubated at 37.5°C (Experiment 2). Yolk sac tissue samples were collected from embryonic d 7 (E7) to day of hatch (DOH). Formalin-fixed paraffin-embedded yolk sac tissue samples (*n* = 3) were processed for in situ hybridization using a probe for chicken occludin and the RNAscope 2.5 HD Assay-RED detection kit. Occludin mRNA was revealed as red fluorescence. Sections were counterstained with hematoxylin. Replicate 1 (Rep1) and Rep2 were hybridized at the same time, while Rep3 was done separately. Fluorescent images were captured at 200× with a Nikon Eclipse 80i microscope and a Nikon DS-Ri1 camera. Scale bar equals 100 μm.

To further investigate the temporal expression pattern of TJ mRNA, ISH was performed using a probe for OCLN as a representative TJ protein. In the YST, OCLN mRNA was expressed highly from E7 to E13 or E11 in Replicates 1 and 2, respectively (Figure 5). In Replicate 3, OCLN mRNA showed high expression at E7 and low expression at other time points. In general, OCLN mRNA was expressed the greatest at the early embryonic ages and lower during late embryogenesis and DOH, which was contrary to the increase in expression at DOH as determined by qPCR.

**Figure 6:**
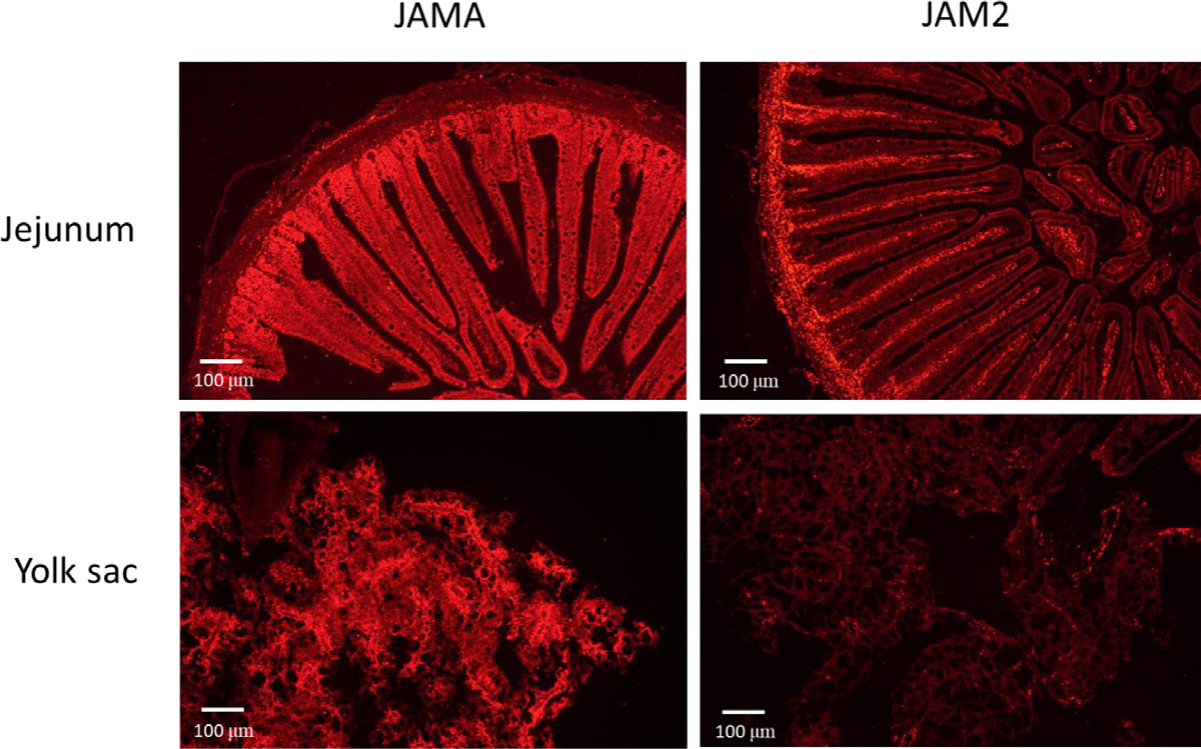
In situ hybridization for JAMA and JAM2 mRNA in the jejunum and yolk sac tissue of day of hatch chicks. Eggs were incubated at 37.5°C (Experiment 1). Sections of the jejunum and yolk sac tissue from chicks at day of hatch were fixed in formaldehyde and embedded in paraffin. In situ hybridization was performed using probes for chicken JAMA and JAM2 and the RNAscope 2.5 HD Assay-RED. JAMA and JAM2 mRNA were revealed as red fluorescence. Sections were counterstained with hematoxylin. Fluorescent images were captured at 100× with a Nikon Eclipse 80i microscope and a Nikon DS-Ri2 camera. Scale bar equals 100 μm.

Two members of the JAM family, JAMA and JAM2, are often used for assessing barrier integrity. In situ hybridization was used to identify cells expressing JAMA and JAM2 mRNA in both the small intestine and YST at DOH (Figure 6). In the jejunum, JAMA mRNA was strongly expressed in the epithelial cells of the villi and crypt. By contrast, JAM2 mRNA was strongly expressed in the center of the villi and in the lamina propria, where the vasculature is located. In the YST, JAMA mRNA was strongly expressed in epithelial cells, whereas JAM2 mRNA showed only scattered light staining. These results demonstrate that JAMA mRNA is highly expressed in the epithelial cells of the villi, crypt and YST, whereas JAM2 mRNA is not expressed in epithelial cells, but in the vascular cells of the villi and lamina propria.

## DISCUSSION

Because the YST is derived from the midgut of the embryonic intestine, these tissues share structural similarities, such as a single layer of epithelial cells that line the yolk sac and intestinal villi. This layer of epithelial cells acts as a physical barrier to regulate the passage of macromolecules and pathogens present in the yolk or intestinal lumen. In this way, pathogens can be prevented from entering the underlying blood system and infecting the host. Thus, a functional TJ between adjacent epithelial cells is essential to seal the intercellular space and prevent passage of unwanted molecules and pathogens.

Mobbs and McMillan (1979) previously showed the presence of TJ in the *area pellucida* and *area vasculosa* of the YS by electron microscopy. In the current study, ZO1, OCLN, CLDN1, JAMA, and JAM2 mRNA were all shown to be expressed in the YST using qPCR. Furthermore, using OCLN and JAMA as representative TJ proteins, ISH analysis confirmed that these 2 genes were strongly expressed in the YST. Although ISH for other TJ proteins was not performed, it is likely that they also are expressed in the epithelial cells of the YST. Together the electron microscopy, qPCR and ISH results demonstrate that the YST like the small intestine contains tight junction complexes.

The observed quadratic profile of TJ mRNA in the YST was repeatable. The high expression of TJ mRNA during early embryogenesis would coincide with the time that the YST is developing multiple physiological functions such as erythropoiesis, host defense, nutrient uptake, and metabolic regulation (Wong and Uni, 2021). Similar increases in mRNA during late embryogenesis for some amino acid and monosaccharide transporters in the YST of chicks prior to hatch have been reported (Speier et al., 2012). This increase in mRNA expression during late embryogenesis was also observed in the YS membrane (same as the YST) of catshark embryos (Honda et al., 2022). These researchers found increased mRNA abundance of genes involved in amino acid transport, lipid metabolism and lysosomal digestion. These results indicate that the YST is still functionally active at hatch and expresses genes important for metabolic processes and maintaining epithelial barrier integrity.

In situ hybridization is a valuable tool for identifying cells expressing an mRNA of interest and as an alternative method of assessing gene expression as a complement to qPCR. ISH was used to demonstrate that OCLN mRNA did not increase in the YST with embryonic age as indicated by qPCR. Although it is not clear why there is a discrepancy, in our experience, small changes in gene expression by qPCR are typically not observable by ISH. In addition, ISH was used to show that JAMA mRNA was expressed in the epithelial cells of the intestinal villi and crypt as well as the YST, whereas JAM2 was expressed in the vasculature of the villi and the lamina propria. This expression pattern in the small intestine of DOH chicks is the same as that previously reported in the small intestine of 21-day-old broiler chicks (Wong and Kinstler, 2023). Thus, JAMA mRNA is the correct gene to use when assessing intestinal barrier integrity, while JAM2 mRNA would be appropriate for assessing barrier function in vascular tissue.

High incubation temperature increased embryonic mortality and reduced hatchability and yolk utilization of broiler chickens (reviewed in van der Wagt et al., 2020). However, these effects mainly focused on the late stages. Dayan et al. (2020) reported that incubation at 39.3°C caused death mainly between E19 and E21, reducing hatchability to 8.4%. Similar results were also found during incubation of turkey eggs (French, 2000). In the current study, incubation at 39.5°C from E12 to DOH reduced hatchability to 42%, compared with 93% at 37.5°C.

Because the YST is derived from the embryonic intestine, it was of interest to determine if elevated incubation temperature had a similar effect on gene expression in both tissues. High incubation temperature has been previously reported to affect development or gene expression of the YST. Dayan et al. (2020) showed that high incubation temperature (39.3°C vs. 37.8°C from E0 to E18) altered the temporal patterns of mRNA abundance for genes related to lipid uptake and metabolism, oligopeptide uptake, gluconeogenesis, and thyroid hormone regulation in the YST. In our study, incubation at 39.5°C increased ZO1 and JAM2 mRNA in both the YST and small intestine. ZO1 localizes near the cytoplasmic membrane and serves as an important link between the actin cytoskeleton and the TJ, which includes the transmembrane JAM proteins (Zihni et al., 2016; Awad et al., 2017). ZO1 is required for normal blood vessel formation in the yolk sac of mice (Katsuno et al., 2008) and is known to interact with JAM2 (Ebnet et al., 2003). Lin et al. (2017) showed that incubation at 38.1°C increased the vasculature of the YS membrane (same as the YST in our study) in chick embryos at E7. Our results show that JAM2 mRNA is expressed in the vasculature. Thus, it appears that increased incubation temperature upregulated the expression of genes involved in the vascular system of the YST.

Although representing a very different heat stress model, increased environmental temperature of posthatch chickens affected mRNA abundance of TJ proteins in the small intestine. Song et al. (2014) reported that heat stress from d 22 to d 42 by exposure to 33°C for 10 h/d decreased the jejunal protein expression level of OCLN and ZO1. Zhang et al. (2017) also showed similar results. Exposure of 40-wk-old laying hens to 33°C over 10 d or 20 d decreased the ileal expression level of OCLN and ZO1 mRNA. By contrast, in our study, there were increases of ZO1 and JAM2 mRNA in the small intestine at 39.5°C. Thus, for the embryonic chicks, the increase in TJ mRNA may enhance structural integrity of the epithelial barrier of the small intestine, whereas in the posthatch chick the decrease in TJ mRNA may lead to a weakening of the epithelial barrier, perhaps leading to leaky gut syndrome.

In conclusion, the YST expresses ZO1, OCLN, CLDN1, and JAMA mRNA, which are important for maintaining the tight junction between epithelial cells that line the YST, similar to that of the small intestine. High incubation temperature (39.5°C) from E12 to DOH affected the expression of ZO1 and JAM2 mRNA in both the YST and embryonic small intestine. This suggests that the TJ in the vasculature of the YST and small intestine is affected by high incubation temperature.
